# Clinical Impact of Sequence Type 131 in Adults with Community-Onset Monomicrobial *Escherichia Coli* Bacteremia

**DOI:** 10.3390/jcm7120508

**Published:** 2018-12-03

**Authors:** Jiun-Ling Wang, Ching-Chi Lee, Chung-Hsun Lee, Nan-Yao Lee, Chih-Chia Hsieh, Yuan-Pin Hung, Hung-Jen Tang, Wen-Chien Ko

**Affiliations:** 1 Department of Internal Medicine, National Cheng Kung University Hospital, College of Medicine, National Cheng Kung University, Tainan 70403, Taiwan; jiunlingwang@gmail.com (J.-L.W.); chichingbm85@gmail.com (C.-C.L.); nanyao@mail.ncku.edu.tw (N.-Y.L.); yuebin16@yahoo.com.tw (Y.-P.H.); 2 Division of Critical Care Medicine, Department of Internal Medicine, Madou Sin-Lau Hospital, Tainan 72152, Taiwan; 3 Graduate Institute of Medical Sciences, College of Health Sciences, Chang Jung Christian University, Tainan 71101, Taiwan; 4 Department of Emergency Medicine, National Cheng Kung University Hospital, College of Medicine, National Cheng Kung University, Tainan 70403, Taiwan; chlee82er@yahoo.com.tw (C.-H.L.); hsiehchihchia@gmail.com (C.-C.H.); 5 Department of Internal Medicine, Tainan Hospital, Ministry of Health and Welfare, Tainan 70043, Taiwan; 6 Department of Medicine, Chi-Mei Medical Center, Tainan 71004, Taiwan; 8409d1@gmail.com (H.-J.T.); 7 Department of Health and Nutrition, Chia Nan University of Pharmacy and Science, Tainan 71710, Taiwan

**Keywords:** ST131, ESBL, bacteremia, *Escherichia coli*, mortality

## Abstract

Background: The clinical impact of ST (sequence type) 131 in adults with community-onset *Escherichia coli* bacteremia remains controversial. Methods: Clinical data of 843 adults presenting with community-onset monomicrobial *E. coli* bacteremia at a medical center between 2008 and 2013 were collected. *E. coli* isolates were genotyped by a multiplex polymerase chain reaction to detect ST131 and non-ST131 clones. Results: Of 843 isolates from 843 patients with a mean age of 69 years, there were 102 (12.1%) isolates of ST131. The ST131 clone was more likely to be found in the elderly (76.5% vs. 64.0%; *p* = 0.01) and in nursing-home residents (12.7% vs. 3.8%; *p* < 0.001) than non-ST131 clones. Furthermore, the ST131 clone was associated with a longer time to appropriate antibiotic therapy (2.6 vs. 0.8 days; *p* = 0.004) and a higher 28-day mortality rate (14.7% vs. 6.5%, *p* = 0.003). In the Cox regression analysis with an adjustment of independent predictors, the ST131 clone exhibited a significant adverse impact on 28-day mortality (adjusted odds ratio (aOR), 2.18; *p* = 0.02). The different impact of the ST131 clone on 28-day mortality was disclosed in the non-ESBL (aOR 1.27; *p* = 0.70) and ESBL (aOR 10.19; *p* = 0.048) subgroups. Conclusions: Among adults with community-onset *E. coli* bacteremia, the ST131 clone was associated with higher 28-day mortality, particularly in those infected by ESBL producers.

## 1. Introduction

Bacteremia is significantly associated with significant healthcare costs and high fatality [1,2]. *Escherichia coli* is the leading pathogen of community-onset bacteremia resulting from urosepsis, intra-abdominal infection, or biliary tract infections [3,4]. The emergence of multidrug resistance (MDR) among clinical *E. coli* isolates may lead to inappropriate empirical antibiotic therapy and an unfavorable outcome [5,6].

Since 2000, the sequence type (ST) 131 clone has caused a global epidemic of extended-spectrum β-lactamase (ESBL)-producing and fluoroquinolone-resistant *E. coli* infections [7,8]. The acquisition of MDR determinants among the ST131 clone limits the use of third-generation cephalosporins and fluoroquinolones as an empirical therapy. The successful dissemination and expansion of the key ST131 clone may be explained by a better ability to colonize and persist in the intestine or urinary tract, greater virulence, and more extensive antimicrobial resistance compared to other *E. coli* clones [9].

There have been a few studies discussing the risk factors and prognosis of *E. coli* ST131 bloodstream infections [10,11,12], and these studies mainly focused on ESBL-producing *E. coli* infections. In previous reports of ESBL-producing *E. coli* bacteremia from Taiwan, Spain, and Korea, ST131 was not found to be associated with a higher mortality rate [10,11,12]. Focusing on the overall bacteremia, the data of the clinical picture and prognosis of ST131 *E. coli* bacteremia in comparison with other clones were lacking. Accordingly, a clinical study dealing with adult patients having community-onset *E. coli* bacteremia was conducted to disclose the clinical impact of the ST131 clone.

## 2. Materials and Methods

### 2.1. Study Design and Population

A retrospective cohort study was conducted at a medical center in southern Taiwan. The institutional review board of the study hospital approved the investigation (A-ER-104-313), which was reported in the format recommended by STROBE (Strengthening the Reporting of Observational Studies in Epidemiology) [13].

Of the blood cultures sampled from adults in the emergency department (ED) between January 2008 and December 2013, culture results were screened in a computer database. The clinical information of adults with monomicrobial *E. coli* bacteremia was retrieved from medical records and recorded in a predetermined form. If multiple bacteremic episodes were noted in one patient, only the first episode was included. Patients were excluded if they had incomplete clinical data, hospital-onset bacteremia, or uncertain outcome information in medical records.

### 2.2. Data Collection

For eligible patients, demographic and clinical characteristics, including age, initial syndrome, vital signs at the ED, comorbidities, laboratory data, duration and type of antimicrobial therapy, bacteremia source, hospital stay, bacteremia severity (Pitt bacteremia score), comorbidity severity (McCabe classification), and patient outcome were collected. The medical records were independently reviewed by two authors, and any discrepancy was solved by discussion between the authors.

The primary endpoint was the crude mortality within 28 days after bacteremia onset. The times to defervescence and crude mortality were regarded as outcome variables. To avoid underestimating the mortality rate, if a patient was discharged earlier than 28 days after bacteremic onset and was not followed up at the outpatient clinic, outcome information was collected by telephone contact. Patients unable to be reached by telephone were excluded.

### 2.3. Microbiological and Molecular Methods

Blood cultures were incubated in the BACTEC 9240 instrument (Becton Dickinson Diagnostic Systems, Sparks, MD, USA) for 5 days at 35 °C, and *E. coli* was identified using a GNI (Identification of Gram-negative bacilli) Card from the Vitek system (bioMerieux, Lyon, France). The susceptibility was determined by the broth microdilution method in a Vitek 2 (bioMerieux, Durham, NC, USA) automated susceptibility system, according to the contemporary breakpoints recommended by the Clinical and Laboratory Standards Institute (CLSI) in 2016 [14]. ESBL production was examined by the phenotypic confirmatory test with cephalosporin-clavulanate combination disks, as recommended by the CLSI [15]. Several studies have shown the predominance of four major lineages (ST69, ST73, ST95, and ST131) among international extra-intestinal pathogenic *E. coli* collections of human infection and 45–58% of bacteremia and urine samples [7,16]. A multiplex polymerase chain reaction (PCR) described by Doumith et al. was used to distinguish the ST131 from the non-ST-131 clone, to avoid the time and cost associated with traditional MLST (Multilocus sequence typing) [17,18]. This multiplex PCR is quite specific to ST131, but not specific to ST69, ST73, and ST95 because this PCR can also detect single-locus variants (SLVs) [17]. Most disturbing is the fact that the false-positive results are not always related to SLVs, but sometimes to a different phylogroup [19].

### 2.4. Definitions

Community-onset bacteremia indicate that the place of bacteremia onset is the community, and it includes nursing-home-associated and community-acquired bacteremia [4,20]. Patients transferred from other hospitals to EDs were regarded as having hospital-onset bacteremia. As previously described [4,20], antimicrobial therapy was considered to be appropriate when both of the following criteria were fulfilled: (i) the route and dosage of antimicrobial agent(s) administered were as recommended in the Sanford Guide [21]; and (ii) bacteremic pathogens were in vitro susceptible to the administrated antimicrobial agent(s) based on the contemporary CLSI breakpoints [14]. The time taken to appropriate antibiotic therapy measured in hours was the period between arrival at the ED (i.e., bacteremia onset) and the administration of appropriate antimicrobials. Inappropriate empirical therapy was defined as the time to appropriate antibiotic therapy of > 48 h [4].

Malignancy included hematological malignancies and solid tumors. Comorbidities were defined as described previously [22]. The prognosis of preexisting diseases was assessed by a previous delineated classification system (McCabe classification) [23]. The sources of bacteremia were determined by being clinically based on the presence of an active infection site coincident with bacteremia, or the isolation of a microorganism from other clinical specimens prior to or on the same date of bacteremia onset. If the bacteremia source could not be assigned to a specific site, it was classified as primary bacteremia. Crude mortality was used to define death from all causes, whereas the death of a patient with a clinical course suggestive of a persistently active infection without an obvious explanation was referred to as sepsis-related mortality. The Pitt bacteremia score—a validated scoring system based on vital signs, usage of inotropic agents, mental status, receipt of mechanical ventilation, and recent cardiac arrest—was utilized to grade the severity of bacteremia [4]. Patients with a high Pitt bacteremia score (≥ 4) were graded as having a critical illness, whereas those with a low Pitt bacteremia score (= 0) were graded as stable.

### 2.5. Statistical Analysis

Statistical analyses were performed using the Statistical Package for the Social Sciences for Windows (Version 20.0, Chicago, IL, USA). In the case of missing values, a complete case analysis was conducted if the missing values were below 5%, and thus an analysis might be feasible in that case. If the missing values were at or above 5%, the appropriate imputation was performed. Clinical data, demographic data, severity, and patient outcomes were compared by the Fisher exact or Pearson Chi-square test for categorical variables, and an independent *t* or Mann-Whitney test for continuous variables. The variables associated with 28-day mortality in the univariate analysis with a *p* value less than 0.05 were included in a stepwise and backward multivariable logistic regression model to evaluate their independent effects on the clinical outcome. To compare the effects of ST131 and non-ST131 clones on patient survival and the impact of ESBL production on patient prognosis, Kaplan–Meier survival curves and Cox regression were assessed after adjustment of independent predictors of 28-day mortality. A two-sided *p* value less than 0.05 was considered significant.

## 3. Results

### 3.1. Demographics and Clinical Characteristics of the Study Population

A total of 843 adults with monomicrobial *E. coli* bacteremia were included; their mean age was 69.2 years, and 551 (65.4%) were female. Common comorbidities included hypertension (443 patients, 52.6%), diabetes mellitus (315, 37.4%), malignancy (211, 25.0%), neurological disorder (167, 19.8%), chronic kidney disease (123, 14.6%), liver cirrhosis (103, 12.2%), coronary artery disease (85, 10.1%), congestive heart failure (57, 6.8%), urological disease (46, 5.5%), chronic obstructive pulmonary diseases (37, 4.4%), and autoimmune disease (18, 2.1%). The leading sources of bacteremia were urinary tract infections (527 patients, 62.5%), biliary tract infections (104, 12.3%), intra-abdominal infections (103, 12.2%), pneumonia (38, 4.5%), and soft-tissue infection (12, 1.4%). Primary bacteremia without a clinically recognized infectious focus was noted in 41 (4.9%) patients.

Of the 843 adults, their median (interquartile range (IQR)) ED stay was 13.8 (4.9–23.8) h. Most (731, 86.7%) patients were admitted to general wards, 54 (6.4%) to intensive care units (ICUs), and 14 (1.7%) died during the ED stay. The median (IQR) length of hospital stay was 8 (6–13) days. Only 44 (5.2%) were discharged from the ED and then followed up in outpatient clinics. The proportion of critically ill patients (i.e., Pitt bacteremia score ≥ 4) and 28-day crude mortality were 13.5% (114 patients) and 7.5% (63), respectively.

### 3.2. Comparisons of Clinical Characteristics, Severity, Susceptibility, and Outcome Between ST131 and Non-ST131 Clonal Groups

**Figure 1:**
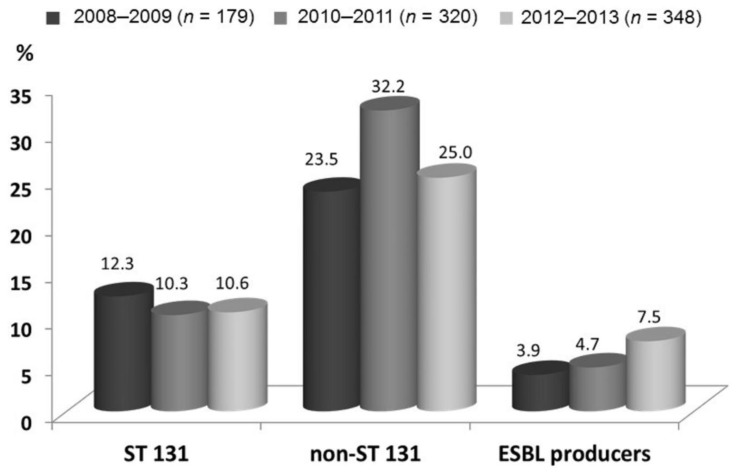
Proportions of sequence type (ST) 131 and non-ST131 clones and extended-spectrum β-lactamase (ESBL) producers in community-onset *Escherichia coli* bacteremia in three periods from 2008 to 2013. An annual increasing trend in ESBL producers was observed (*γ* = 1.00, *p* = 0.01).

Of 324 (38.4%) typable isolates, there were 102 (12.1%) isolates of the ST131 clone. The percentages of ST131 and non-ST131 clones and ESBL producers during the study period are shown in Figure 1. Notably, of 48 ESBL producers, more than one-half (58.3%) were ST131 isolates. A positive annual trend in ESBL production was noted (*γ* = 1.00, *p* = 0.01), but not in the ST131 clone (*γ* = −0.50, *p* = 0.67).

Clinical characteristics of 102 adults infected by the ST131 clone were compared with 741 by non-ST131 isolates in Table 1. Fewer females (53.9% vs. 66.9%; *p* = 0.01), more elderly (76.5% vs. 64.0%; *p* = 0.01) or nursing-home residents (12.7% vs. 3.8%; *p* < 0.001), and more comorbidities with urological (13.7% vs. 4.3%; *p* < 0.001) or neurological (28.4% vs. 18.6%; *p* = 0.02) diseases were evident in those with ST131 bacteremia. Lower susceptibility rates of cefazolin (7.8% vs. 51.3%; *p* < 0.001), cefotaxime (64.7% vs. 91.5%; *p* < 0.001), cefepime (90.2% vs. 98.1%; *p* < 0.001), ertapenem (96.1% vs. 99.3%; *p* = 0.02), or levofloxacin (62.7% vs. 90.1%; *p* < 0.001) and more ESBL producers (27.5% vs. 2.7%; *p* < 0.001) were present in ST131 isolates.

However, the proportions of a critical illness at bacteremia onset, initial presentation with severe sepsis or septic shock, and comorbidity severity were similar between the ST131 and non-ST131 groups (Table 2). Of note, a longer time to appropriate antibiotic therapy (mean; 2.6 vs. 0.8 days; *p* = 0.004) and higher crude 14-day (10.8% vs. 4.3%; *p* = 0.005) and 28-day (14.7% vs. 6.5%; *p* = 0.003) mortality rates were present in the ST131 group. 

In the ESBL-producing isolates, the percentages of inappropriate antibiotic therapy in the ST131 and non-ST131 group were similar (71.4% vs. 65.0%; *p* = 0.64), but the ST131 group had higher crude 14-day (21.4% vs. 10.0%; *p* = 0.44) and 28-day (35.7% vs. 20.0%; *p* = 0.34) mortality rates than the non-ST131 group. For non-ESBL producers, inappropriate antibiotic therapy was more common in the ST131 group (18.9% vs. 7.6%; *p* = 0.001), but the crude 14-day (6.8% vs. 4.2%; *p* = 0.36) and 28-day mortality (6.8% vs. 6.1%; *p* = 0.82) were similar in the ST131 and non-ST131 groups.

Susceptibility data of the ST131 and non-ST131 clonal isolates in ESBL producers and non-ESBL producers are listed in Table 3. In non-ESBL-producing *E. coli* isolates, ST131 isolates were less susceptible to cefazolin, ertapenem, and levofloxacin than non-ST131 isolates. However, among 48 ESBL-producing *E. coli* isolates, the susceptibility rates of five antimicrobial agents were similar between ST131 and non-ST131 isolates (Table 3).

### 3.3. Risk Factors of 28-Day Crude Mortality

In the univariate analyses, 28-day mortality was positively associated with nursing-home residence, inappropriate empirical antibiotic therapy, critical illness (a Pitt bacteremia score ≥ 4) at bacteremia onset, primary bacteremia, bacteremic pneumonia, intra-abdominal infection, soft-tissue infection, fatal comorbidity (McCabe classification), and underlying malignancy or liver cirrhosis (Table 4). In contrast, being female, urosepsis, and underlying hypertension or diabetes mellitus were negatively linked to 28-day mortality. In the multivariate regression analyses, only seven independent predictors were identified, including nursing-home residence, critical illness at bacteremia onset, inappropriate empirical therapy, a fatal comorbidity, underlying liver cirrhosis, and specific bacteremia sources (i.e., pneumonia or soft-tissue infection).

### 3.4. Clinical Impact of the ST131 Clone and ESBL Production

**Figure 2:**
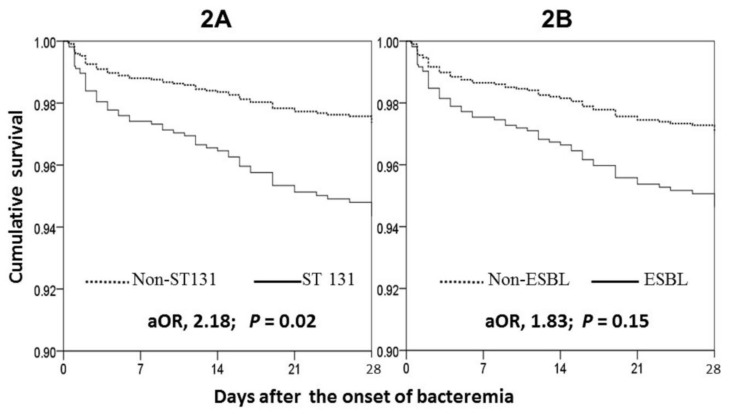
Survival curves of the cases of community-onset *Escherichia coli* bacteremia due to ST131 or non-ST131 isolates (**2A**), and the isolates with or without ESBL production (**2B**) in the Cox regression after adjustment of independent predictors of 28-day mortality *. aOR: adjusted odds ratio. * Includes fatal comorbidity (McCabe classification), Pitt bacteremia score ≥ 4 at bacteremia onset, inappropriate empirical antimicrobial therapy, bacteremia due to pneumonia or soft-tissue infections, underlying liver cirrhosis, and nursing home residence.

The Kaplan-Meier survival curve showed a significant difference in 28-day survival between adults infected with ST131 and non-ST131 *E. coli* bacteremia (adjusted odds ratio (aOR), 2.18; 95% confidence interval (CI), 1.16–4.10; *p* = 0.02) (Figure 2A), but no difference between those with ESBL producers and non-ESBL producers (aOR, 1.83; 95% CI, 0.81–4.17; *p* = 0.15) (Figure 2B), after adjustment of all independent prognostic variables linked to 28-day crude mortality, as listed in Table 4.

### 3.5. Clinical Impact of the ST131 Clone in the ESBL and Non-ESBL Subgroups

Of 795 patients with non-ESBL-producing *E. coli* bacteremia (the non-ESBL group), the independent factors recognized in the multivariate regression included comorbid malignancy or liver cirrhosis, fatal comorbidity (McCabe classification), critical illness at bacteremia onset, and bacteremia due to pneumonia or soft-tissue infection (Table 5). In the univariate or multivariate analyses, the ST131 clone was not associated with 28-day mortality. However, of 48 patients with ESBL-producing *E. coli* bacteremia (the ESBL group), in addition to nursing-home residence and a critical illness at bacteremia onset, the ST131 clone was independently associated with 28-day mortality in the multivariate regression (Table 5).

**Figure 3:**
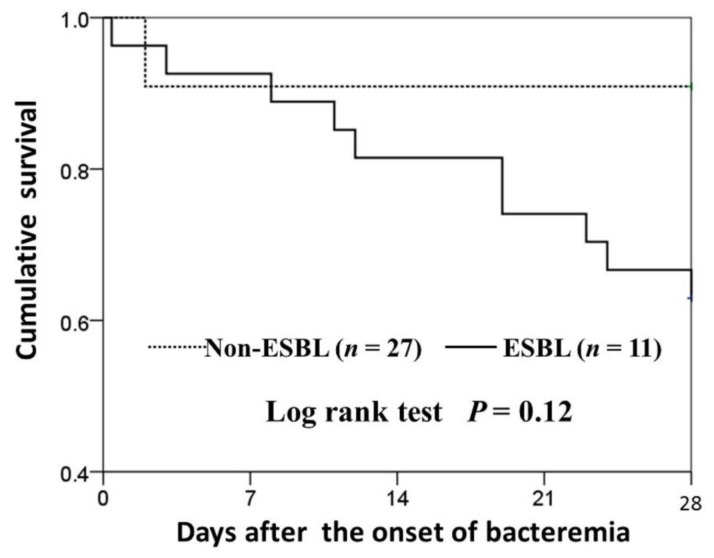
Survival curves of the cases of community-onset bacteremia due to levofloxacin-resistant ST131 *Escherichia coli*, categorized by the presence or absence of ESBL production.

For the levofloxacin-resistant ST131 isolates, survival curves revealed a higher 28-day mortality rate in the ESBL producers (10/27, 37.0%) than in the non-ESBL-producers (1/11, 9.1%), but the difference was not statistically significant (*p* = 0.12) (Figure 3).

**Figure 4:**
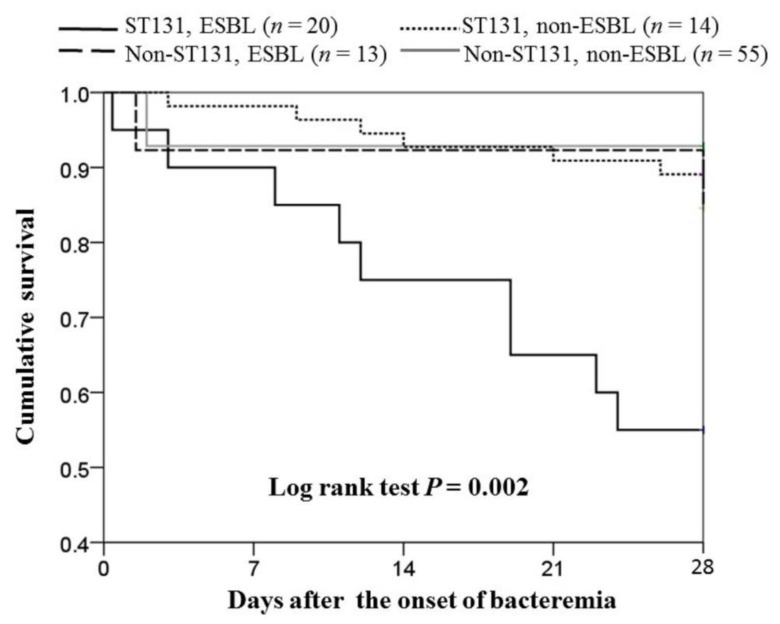
Survival curves of the cases of community-onset ST131 and non-ST131 *Escherichia coli* bacteremia empirically treated by an inappropriate antibiotic, categorized by the presence or absence of ESBL production.

For 28-day crude mortality rates among the cases of community-onset bacteremia due to ST131 and non-ST131 *E. coli* isolates with or without ESBL production treated by inappropriate empirical antimicrobials, those infected by ST131 isolates with ESBL production had the highest mortality rate (Figure 4).

## 4. Discussion

In this large cohort of community-onset *E. coli* bacteremia, the ST131 clone was more likely to cause bacteremia in the elderly and those with underlying urological diseases, and was associated with antimicrobial resistance, delayed appropriate treatment, longer hospital stays, and subsequently, a worse outcome. Notably, in comparison with other potential prognostic microbiological characteristics, such as ESBL production, the adverse impact of the ST131 clone on a short-term prognosis was disclosed in our population. The finding that ST131 isolates were often present in elderly or nursing-home residents corresponded to recent reports that ST131 has spread in nursing homes in Europe and the U.S. [24,25,26,27]. More underlying urological disorders were found in affected individuals with ST131 infections here. This implies that ST131 isolates tend to colonize or infect compromised hosts, especially those with urinary tract abnormalities [27]. The emergence of ST131 in urological patients may make empirical antibiotics difficult, since there was increasing fluoroquinolone or extended-spectrum beta-lactam resistance in ST131 isolates [28,29].

In previous studies of ESBL-producing *E. coli* bacteremia, the ST131 clone was not independently associated with mortality [10,11,12]; further, in two published studies comparing small numbers of bacteremic events due to non-ESBL-producing ST131 and non-ST131 clones, the ST131 clone did not show an association with a worse outcome [9,30]. However, in two studies of urinary tract infections, the ST131 clone was a significant risk factor for treatment failure [27,31]. Furthermore, the H30 subclone of ST131 was associated with clinical persistence, microbiological persistence, and subsequent new infections, as well as with older, compromised, and functionally impaired hosts [27]. In contrast to previous reports, our cohort including both non-ESBL-producing and ESBL-producing isolates exhibited the adverse prognostic impact of the ST131 clone among adults with community-onset bacteremia. The strengths of our work were the large number of cases examined and the inclusion of non-ESBL-producing isolates, which may partially explain the different result.

With the existence of prognostic significance of ST131 isolates, the virulence potential of the ST131 clone warrants investigation. In a murine model, ST131 isolates did not exhibit higher virulence potential than other *E. coli* isolates, and extensive within-ST virulence diversity was found in two murine studies [32,33]. Notably, among five extra-intestinal pathogenic *E. coli* STs (i.e., ST131, ST95, ST73, ST69, and ST127), the *Galleria mellonella* model showed that ST131 was significantly more virulent than other STs [34]. Using another animal model, the zebra fish lethality assay, the ST131 isolates were also found to be more lethal than non-ST131 isolates [35]. Despite these equivocal findings diminishing the practicality of ST131 clonal detection, the clinical importance of ST131 clonal detection was highlighted in our cohort.

Similar to previous studies examining the outcome of ESBL production in community-onset *E. coli* bacteremia [36,37], our cases of bacteremia due to ESBL producers and non-ESBL producers had similar survival rates at 28 days, as noted in the multivariate regression model after adjustment of all independent predictors linked to mortality. Moreover, the adverse impact of the ST131 clone still exists after adjustment of the same variables. However, when focusing on fluoroquinolone-resistant ST131 isolates, we found that the effect of ESBL producers on mortality was not evident. A distinct sublineage of ST131, H30-Rx, with *bla*_CTX-M-15_ and fluoroquinolone resistance can be separated from ESBL-negative fluoroquinolone-resistant H30 isolates by three core genome single-nucleotide polymorphisms [38], indicating that ST131 isolates are not genetically homogenous; however, more studies on the prognostic impact of the subgroups of ST131 isolates are needed.

This study was limited to the data from a single center in Taiwan. The genetic relationship of the ST131 isolates was not compared with others in the world, though a report from Taiwan found more CTX-M14 than CTX-M15 in ESBL-producing *E. coli* ST131 isolates [11]. The multiplex PCR reaction can detect only major lineages of *E. coli* ST isolates, and the clinical role of other STs, accounting for at least 60% of our bacteremic isolates, is not known. The overall percentage of four clones in this study (38%) is similar to that found in surveillance data in England [19]. Using the PCR method proposed by Doumith et al. to detect four STs (ST69, ST95, ST73, and ST131) may lead to false positive results, especially for ST69 and ST95. This makes the analysis and the results based on this PCR for ST69 and ST95 incorrect, but this can be resolved by traditional MLST typing or whole-genome sequencing [19]. Luckily, PCR is quite specific for ST131. Thus, our included *E. coli* isolates were grouped as ST131 and non-ST131 clones, rather than four distinct STs.

## 5. Conclusions

For adults with community-onset *E. coli* bacteremia, the ST131 clone has been found to be related to specific host characteristics, such as preference for the elderly and those with underlying urological diseases, and indicates a worse prognosis, particularly for the isolate with ESBL production.

## Figures and Tables

**Table 1:** Clinical characteristics of adults with community-onset monomicrobial *Escherichia coli* bacteremia due to sequence type 131 (ST131) and non-ST131 isolates.

Characteristics	Patients Number (%)		*p*-Values
	ST131, *n* = 102	Non-ST131, *n* = 741	
Gender, female	55 (53.9)	496 (66.9)	0.01
The elderly, ≥65 years	78 (76.5)	474 (64.0)	0.01
Nursing home residents	13 (12.7)	28 (3.8)	<0.001
In vitro susceptibility			
Cefazolin-S	8 (7.8)	380 (51.3)	<0.001
Cefotaxime-S	66 (64.7)	678 (91.5)	<0.001
Cefepime-S	92 (90.2)	727 (98.1)	<0.001
Ertapenem-S	98 (96.1)	736 (99.3)	0.02
Levofloxacin-S	64 (62.7)	668 (90.1)	<0.001
ESBL producers	28 (27.5)	20 (2.7)	<0.001
Major comorbidity			
Hypertension	54 (52.9)	389 (52.5)	0.93
Diabetes mellitus	38 (37.3)	277 (37.4)	0.98
Neurological disease	29 (28.4)	138 (18.6)	0.02
Malignancy	28 (27.5)	183 (24.7)	0.55
Coronary artery disease	15 (14.7)	70 (9.4)	0.10
Chronic kidney disease	14 (13.7)	109 (14.7)	0.79
Urological disorder	14 (13.7)	32 (4.3)	<0.001
Liver cirrhosis	13 (12.7)	90 (12.1)	0.86
Major source of bacteremia			
Urinary tract infection	67 (65.7)	460 (62.1)	0.48
Biliary tract infection	12 (11.8)	92 (12.4)	0.85
Intra-abdominal infection	8 (7.8)	95 (12.8)	0.15
Primary bacteremia	7 (6.9)	34 (4.6)	0.32
Pneumonia	4 (3.9)	34 (4.6)	0.76
Soft-tissue infection	1 (1.0)	11 (1.5)	1.00

Boldface indicates statistical significance, i.e., a *p*-value of < 0.05.

**Table 2:** Bacteremia and comorbidity severity and clinical outcomes of adults with community-onset monomicrobial *Escherichia coli* bacteremia due to sequence type 131 (ST131) and non-ST131 isolates.

Characteristics	Patients Number (%)		*p*-Values
	ST131, *n* = 102	Non-ST131, *n* = 741	
Pitt bacteremia score ≥4 at onset	15 (14.7)	99 (13.4)	0.71
Initial sepsis-related syndrome			
Severe sepsis	36 (35.3)	294 (39.7)	0.40
Septic shock	14 (13.7)	106 (14.3)	0.88
Comorbidity severity (McCabe classification)			0.81
Ultimately and rapidly fatal	20 (19.6)	138 (18.6)	
Nonfatal	82 (80.4)	603 (81.4)	
Length, day (mean ± standard deviation)			
Time to appropriate antibiotic	2.6 ± 6.1	0.8 ± 3.8	0.004
Total hospital stays	14.6 ± 17.1	11.1 ± 11.7	0.05
Crude mortality rate			
14 days	11 (10.8)	32 (4.3)	0.005
28 days	15 (14.7)	48 (6.5)	0.003

Boldface indicates statistical significance, i.e., a *p*-value of < 0.05.

**Table 3:** Susceptibility profile* of community-onset bacteremic *Escherichia coli* isolates, categorized by ESBL production and ST131 clone.

Susceptible to Indicated Drugs	Non-ESBL Producers			ESBL Producers		
	ST131 *n* = 74	Non-ST131 *n* = 721	*p*-Values	ST131 *n* = 28	Non-ST131 *n* = 20	*p*-Values
Cefazolin	8 (10.8)	380 (52.7)	<0.001	0 (0)	0 (0)	-
Cefotaxime	66 (89.2)	678 (94.0)	0.13	6 (21.4)	4 (20.0)	1.00
Cefepime	74 (100)	719 (99.7)	1.00	18 (63.4)	8 (40.0)	0.10
Ertapenem	71 (95.9)	719 (99.7)	0.007	27 (96.4)	17 (85.0)	0.29
Levofloxacin	63 (85.1)	668 (96.2)	0.02	1 (3.6)	0 (0)	1.00

* Susceptible isolate number (%). Boldface indicates statistical significance, i.e., a *p*-value of < 0.05.

**Table 4:** Clinical predictors of 28-day mortality in 843 adults with community-onset monomicrobial *Escherichia coli* bacteremia.

Variables	Univariate Analysis		Multivariate Analysis	
	Odds Ratio (95% CI)	*p*-Values	Odds Ratio (95% CI)	*p*-Values
Gender, female	0.48 (0.29–0.81)	<0.001	NS	NS
Nursing home residence	6.98 (3.41–14.31)	0.001	3.86 (1.32–11.31)	0.01
Fatal comorbidity (McCabe classification)	5.76 (3.39–9.79)	<0.001	3.51 (1.63–7.58)	0.001
Inappropriate EAT	3.31 (1.83–5.99)	<0.001	3.02 (1.20–7.59)	0.02
Pitt bacteremia score ≥4 at onset	11.09 (6.41–19.20)	<0.001	17.95 (8.46–38.09)	<0.001
Source of bacteremia				
Urinary tract infection	0.25 (0.14–0.43)	<0.001	NS	NS
Intra-abdominal infection	2.46 (1.32–4.57)	0.003	2.31 (0.98–5.48)	0.06
Primary bacteremia	2.74 (1.16–6.47)	0.03	NS	NS
Pneumonia	5.07 (2.34–10.99)	<0.001	4.55 (1.56–13.26)	0.005
Soft-tissue infection	6.54 (1.91–22.36)	0.009	12.38 (2.41–63.54)	0.003
Comorbidities				
Hypertension	0.31 (0.17–0.55)	<0.001	NS	NS
Diabetes mellitus	0.55 (0.31–0.98)	0.04	NS	NS
Malignancy	3.72 (2.21–6.27)	<0.001	2.00 (0.96–4.20)	0.07
Liver cirrhosis	6.42 (3.69–11.16)	<0.001	4.51 (2.06–9.86)	<0.001

CI: confidence interval; EAT: empirical antibiotic therapy; NS: not significant (after processing the backward multivariate regression).

**Table 5:** Clinical predictors of 28-day mortality in adults with community-onset monomicrobial bacteremia due to *Escherichia coli* with or without ESBL production.

Characteristics	Odds Ratio (95% CI)				
	Non-ESBL Producers, *n* = 795			ESBL Producers, *n* = 48	
	Univariate	Multivariate		Univariate	Multivariate
Gender, female	0.59 (0.33–1.05)	–		0.51 (0.13–1.94)	–
The elderly, ≥65 years	0.71 (0.40–1.27)	–		1.13 (0.25–5.07)	–
ST131 isolates	1.12 (0.43–2.91)	1.27 (0.37–4.41)		2.22 (0.58–8.49)	10.19 (1.02–101.39)
Nursing home residence	3.40 (1.11–10.42)	NS		5.00 (1.32–18.95)	7.09 (1.35–37.13)
Comorbidities					
Liver cirrhosis	7.75 (4.19–14.31)	5.57 (2.49–12.46)		1.87 (0.44–8.01)	–
Malignancy	3.95 (2.20–7.10)	2.47 (1.11–5.49)		1.83 (0.52–0.68)	–
Chronic kidney disease	1.18 (0.54–2.58)	–		0.64 (0.12–3.56)	–
Neurological disease	0.58 (0.24–1.40)	–		2.40 (0.67–6.48)	–
Diabetes mellitus	0.42 (0.20–0.84)	NS		1.07 (0.30–3.78)	–
Hypertension	0.33 (0.17–0.62)	NS		0.35 (0.08–1.47)	–
Fatal comorbidity (McCabe classification)	6.53 (3.60–11.86)	4.20 (1.86–9.49)		2.89 (0.75–11.11)	–
Source of bacteremia					
Soft-tissue infection	8.20 (2.38–28.26)	8.52 (1.64–44.30)		–	–
Pneumonia	4.27 (1.76–10.35)	4.14 (1.45–11.83)		4.10 (2.44–6.84)	NS
Primary bacteremia	3.60 (1.50–8.62)	NS		2.36 (0.14–40.40)	–
Intra-abdominal infection	3.03 (1.57–5.86)	NS		2.54 (0.15–43.67)	–
Biliary tract infection	0.64 (0.23–1.82)	–		0.64 (0.12–3.56)	–
Urinary tract infection	0.18 (0.09–0.35)	NS		0.48 (0.13–1.77)	–
Pitt bacteremia score ≥4 at onset	11.75 (6.36–21.71)	19.93 (8.89–44.64)		10.33 (2.13–50.26)	33.48 (2.92–383.93)
Inappropriate empirical antibiotic therapy	1.84 (0.79–4.27)	–		2.00 (0.47–8.59)	–

NS: not significant (after processing the backward multivariate regression). Boldface indicates statistical significance, i.e., a *p*-value of <0.05.
